# A Novel Active Semisupervised Convolutional Neural Network Algorithm for SAR Image Recognition

**DOI:** 10.1155/2017/3105053

**Published:** 2017-10-01

**Authors:** Fei Gao, Zhenyu Yue, Jun Wang, Jinping Sun, Erfu Yang, Huiyu Zhou

**Affiliations:** ^1^Electronic Information Engineering, Beihang University, Beijing 100191, China; ^2^Space Mechatronic Systems Technology Laboratory, Department of Design, Manufacture and Engineering Management, University of Strathclyde, Glasgow G1 1XJ, UK; ^3^School of Electronics, Electrical Engineering and Computer Science, Queen's University, Belfast BT7 1NN, UK

## Abstract

Convolutional neural network (CNN) can be applied in synthetic aperture radar (SAR) object recognition for achieving good performance. However, it requires a large number of the labelled samples in its training phase, and therefore its performance could decrease dramatically when the labelled samples are insufficient. To solve this problem, in this paper, we present a novel active semisupervised CNN algorithm. First, the active learning is used to query the most informative and reliable samples in the unlabelled samples to extend the initial training dataset. Next, a semisupervised method is developed by adding a new regularization term into the loss function of CNN. As a result, the class probability information contained in the unlabelled samples can be maximally utilized. The experimental results on the MSTAR database demonstrate the effectiveness of the proposed algorithm despite the lack of the initial labelled samples.

## 1. Introduction

Synthetic aperture radar (SAR) has wide applications in both military and civilian fields due to its merits, such as strong penetrating ability and adaption to severe weathers. SAR automatic target recognition technology (SAR-ATR) aims at automatically recognizing the targets from SAR images [1]. With an increasing amount of the data acquired by a SAR imaging system, the SAR-ATR has become one of research hotspots.

Traditional machine learning methods for the SAR-ATR include Support Vector Machine (SVM) [2], local texture feature [3, 4], dictionary learning [5, 6], and sparse representation [7]. These methods have produced some promising results, but they heavily rely on the hand-crafted feature extraction [8]. Because of the imaging nature, clutters and speckling noise exist in the SAR images, which increases the difficulty of feature extraction despite the fact that experts are involved.

In recent years, with the development of deep learning techniques, CNN has received a great attention in object recognition [9–11]. It can automatically extract the target features without experts' intervention. Compared with the traditional machine learning methods, the CNN is more effective and robust and has been successfully applied to SAR image recognition. In [12], a CNN method was proposed for improving the SAR image classification accuracy. The experimental result showed that the CNN method outperforms the Gabor feature extraction-based SVM method, which demonstrated a great potential of the CNN for SAR image recognition. A convolutional network was designed in [13] to automatically extract the features for SAR target recognition. Using the learned convolutional features, the accuracy of 84.7% was achieved on the 10 types of targets in the MSTAR dataset. Zhou et al. [14] studied the application of the Deep Convolutional Neural Networks (DCNN) in the polarimetric SAR image classification, in which the hierarchical spatial features of images could be automatically learned by the DCNN and the classification accuracy was improved significantly.

As can be found, the CNN has made a great breakthrough in the SAR image recognition. However, the sample labelling for SAR image is still time-consuming, and the accuracy of the image recognition decreases quickly when the labelled samples are insufficient. Active learning (AL) can be effective by adding the most informative and reliable unlabelled samples into the labelled training set. As a result, it would be a promising way to solve the above-mentioned problem. Wang et al. proposed an AL method for the SAR image classification based on a SVM classifier [15]. The most uncertain samples were chosen according to the confidence value, and the experimental results showed that the AL-based method can effectively improve the classification accuracy when the labelled samples are insufficient. Babaee et al. presented an active learning method by employing a low-rank classifier as the training model. This method selects the samples whose labels are predicted wrong but the classifier is highly certain about them, namely, the first certain wrong labelled (FCWL) selection criteria [16]. Samat et al. reported an active extreme learning machine (AELM) method for the polarimetric SAR image classification. In this method, the class supports based on the posterior probability are utilized as the selecting criterion. According to the experimental results, the proposed method was faster than the existing techniques in the both learning and classification phases [17].

Active learning method effectively adds the most informative and reliable unlabelled samples into the training set. The remaining samples may be less informative and the use of active learning may cause too much computational complexity. However, the information contained in them can be used to improve the generalization ability of the classification algorithm. Semisupervised learning (SSL) is an effective way to utilize the information contained in the unlabelled samples. The commonly used SSL methods include semisupervised SVM [18], label propagation [19], and semisupervised clustering. Recently, SSL has been successfully applied in the SAR image recognition. Duan et al. introduced a semisupervised classification method incorporating the likelihood space approach in the training and testing processes so that the unlabelled samples can be effectively used to improve the classification performance [20]. To overcome the complexity of data and the difficulty of creating a fine-level ground truth, a semisupervised method for ice-water classification based on self-training was presented in [21]. By integrating the spatial context model, the region merging, and the self-training technique, the proposed algorithm is capable of accurately distinguishing ice and open water in SAR images using a very small number of labelled samples. In [22], the unlabelled samples were analysed by an unsupervised clustering algorithm under the usage of all the available information. Besides, each sample was classified by a supervised method using the available information at the current phase of clustering. The experimental results on the SAR image showed that the proposed semisupervised method leads to promising classification results.

Recently, inspired by the superiority of CNN, AL, and SSL, the combination of the three methods has become a research tendency. For example, a deep active learning method and a semisupervised CNN were constructed [23–25]. The experimental results demonstrated the effectiveness of these methods for hyperspectral or optical image recognition. However, we have seen a number of problems from SAR images, for example, difficult feature extraction, time-consuming sample labelling, and insufficient labelled samples. The developed techniques are rarely applied to SAR image recognition. In this paper, a novel active semisupervised CNN algorithm for SAR image recognition is proposed. First, the most informative and reliable samples selected by the active learning method are labelled using an information entropy criterion. We believe that the information entropy can be used to effectively measure the reliability of the unlabelled samples, and it can be calculated based on the output of the CNN framework. Then, the class probability information of the remaining unlabelled samples is obtained from the output of the softmax layer of the CNN. Afterwards, the class probability information is designed as the regularization term and added to the loss function of the CNN for the retraining purpose. Since the class probability information can effectively control the impact of the unlabelled samples in the training process, the unlabelled samples are well utilized at this stage.

The rest of this paper is arranged as follows. In Section 2, the convolutional neural network is briefly introduced.  Section 3 describes the proposed method in detail. Then experiments are performed in Section 4. Finally, we summarize this paper in Section 5.

## 2. Convolutional Neural Network

As a multilayer neural network structure, CNN is mainly composed of an input layer, a convolution layer, a pooling layer, and an output layer, where both the convolution and pooling layers are hidden. The input layer is used to receive the pixel values from the original image. The convolution layer extracts the image features by utilizing the convolution kernel. The pooling layer uses local image correlation to reduce the amount of data to be processed. The output layer maps the extracted features to the corresponding labels. The training of the CNN is composed of two ways: forward and backward propagation.

### 2.1. Forward Propagation

The mapping process of an image in the CNN is a forward propagation process, where the output of a previous layer is taken as the input of the current layer. In order to provide a full version of the linear model, a nonlinear activation function is added to the neurons of each layer in the mapping process. Since the first layer only receives pixel values from the image, there are no activation functions. From the second layer of the CNN, the nonlinear activation functions are employed. The output of each layer can be expressed as follows:

(1) zl=Wlxl−1+bl,al=σzl,

where *l* denotes the *l*th layer. If *l* = 2, *x*^2−1^ = *x*^1^ is the pixel value matrix of the image. If *l* > 2, *x*^*l*−1^ represents the feature map matrix *a*^*l*−1^, which is extracted from the (*l* − 1)th layer, that is, *x*^*l*−1^ = *a*^*l*−1^ = *σ*(*z*^*l*−1^). *W*^*l*^, *b*^*l*^, and *z*^*l*^ represent the weight matrix, the bias matrix, and the weighted input of the *l*th layer, respectively; *σ* is the nonlinear activation function, and a rectified linear unit (Relu) is selected in this paper. Suppose *l* = *L*; the *L*th layer is the output layer, and *a*^*l*^ denotes the final output vector.

### 2.2. Backpropagation

The standard backward propagation (BP) algorithm is used to update the parameters *W*^*l*^ and *b*^*l*^ of the CNN [10]. The BP algorithm is a supervised learning method which firstly constructs a cost function based on the actual and the expected outputs, and then a gradient descent method (GD) is used to update *W*^*l*^ and *b*^*l*^ along the gradient descent direction of the cost function. In detail, we suppose *E*_0_ represents the cost function of the CNN structure. The error vector of the output layer can be expressed as follows:

(2) $$\begin{array}{c} \delta^{L}=\frac{\partial E_{0}}{\partial z^{L}}. \end{array}$$

In the process of backward propagation, the error vector *δ*^*L*−1^ of the (*L* − 1)th layer can be derived from the error vector *δ*^*L*^ of the output layer. Thus, the error vector *δ*^*l*^ for each layer can be computed by the Chain Rule as follows:

(3) $$\begin{array}{c} \delta^{l}=W^{l+1}\delta^{l+1}\circ \sigma^{\prime }\left(\;z^{l}\;\right), \end{array}$$

where the symbolic ∘ is the Hadamard product (or Schur product) which denotes the element-wise product of the two vectors. The gradients of *W*^*l*^ and *b*^*l*^ are denoted by ∂*E*_0_/∂*W*^*l*^ and ∂*E*_0_/∂*b*^*l*^, respectively. The partial derivative of *E*_0_ to *W*^*l*^ and *b*^*l*^ can be calculated using (1) and (3):

(4) ∂E0∂Wl=∂E0∂al∘∂al∂Wl=δl∘xl−1,∂E0∂bl=∂E0∂al∘∂al∂bl=δl.

The change values of *W*^*l*^ and *b*^*l*^ can be calculated by

(5) ΔWl=−η∂E0∂Wl,Δbl=−η∂E0∂bl,

where *η* represents the learning rate.

### 2.3. The Output Layer

If the number of neurons in the output layer is* N*, the CNN eventually divides the input images into *N* categories. In the forward propagation process, the input of the output layer is *z*_*k*_^*L*^ = *W*_*k*_^*L*^*x*^*L*−1^ + *b*_*k*_^*L*^, *k* ∈ [1,2,…, *N*], since the output of the softmax activation function provides the probability of each class to which a sample belongs. Thus, unlike the middle layer of the CNN, we use the softmax activation function instead of the Relu function in the output layer, which is the key in our proposed method. The output is normalized by the softmax function, which can be expressed as

(6) $$\begin{array}{c} a_{k}=\frac{e^{\theta_{k}z_{k}^{L}}}{\sum_{j=1}^{N}e^{\theta_{j}z_{j}^{L}}}, \end{array}$$

where *a*_*k*_ is the output of the *k*th neuron in the output layer and *θ*_*k*_ is the parameter of the softmax function. It is obvious that ∑_*k*=1_^*N*^*a*_*k*_ = 1, and if one item increases, all the other items will decrease accordingly.

## 3. The Proposed Method

**Figure 1:**
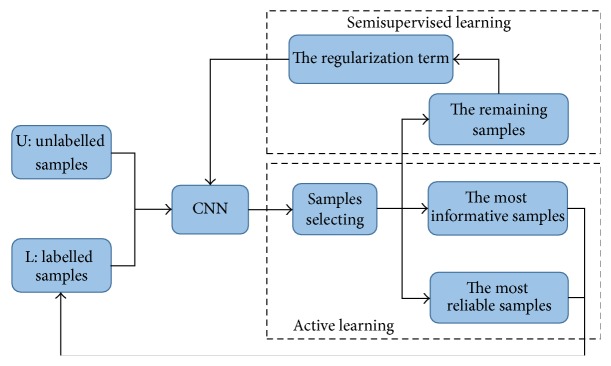
The flowchart of the training process of the proposed algorithm.

First, we define the symbols to be used in this section. The training dataset *X* is composed of two parts: *X* = [*L*, *U*] ∈ *R*^*d*×*N*^, where *L* = [*x*_1_, *x*_2_,…, *x*_*l*_] ∈ *R*^*d*×*l*^ represents the set of the labelled samples and *U* = [*x*_*l*+1_, *x*_*l*+2_,…, *x*_*l*+*u*_] ∈ *R*^*d*×*u*^ represents the set of the unlabelled samples. *l* + *u* = *N* is the total number of the training samples. The training process of the proposed methods is composed of two stages. As shown in Figure 1, first, the most informative and reliable samples selected by the active learning method are labelled based on the information entropy. Then the class probability information extracted from the remaining samples is designed as a regularization term, which will be added to the loss function of the CNN for retraining. When the training process has finished, the unlabelled samples go to the CNN and obtain the labels which can be calculated from the softmax layer of the CNN.

### 3.1. Active Learning

To improve the robustness of the classification model when there are insufficient labelled samples, it is necessary to extend the initial training set. Especially for the CNN model with a large number of parameters, the model will be overfitting and weak in generalization if the labelled samples are inadequate. Thus, an active learning method is utilized to increase the number of training samples.

**Figure 2:**
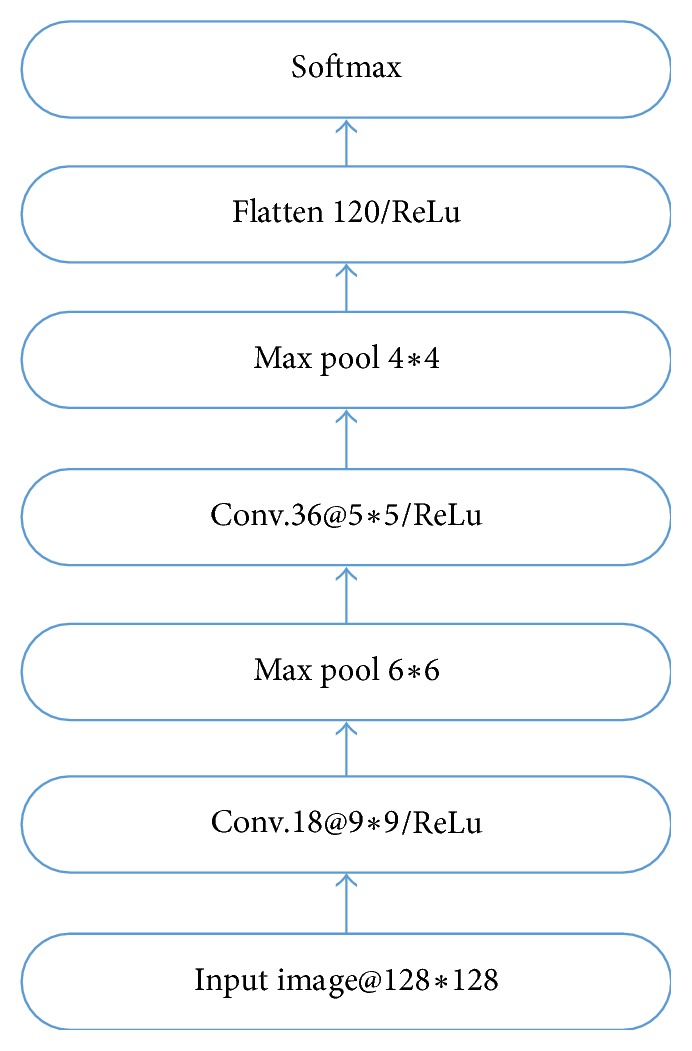
The CNN model employed in this paper.

The CNN model employed in this paper is shown in Figure 2, where Conv, Max pool, and Flatten represent the convolution layer, the subsampling layer, and the fully connected layer, respectively. First of all, CNN is trained using the initially labelled samples. After that, the unlabelled samples go into the CNN, and the information entropy of each unlabelled sample is calculated using the output of the softmax layer. The information entropy measures the uncertainty of the samples. The greater the value of the information entropy is, the greater the uncertainty of a sample is. In other words, if the sample is closer to the classification plane, the probability of the sample belonging to a specific category is more uniform.

The probability of each class to which a sample belongs is represented by [*p*_1_^*i*^, *p*_2_^*i*^,…, *p*_*N*_^*i*^], which can be obtained by (6). Then, the information entropy of the unlabelled sample *x*_*i*_ can be calculated by

(7) $$\begin{array}{c} H^{i}=-\overset{N}{\underset{k=1}{\sum }}p_{k}^{i}\log\left(\;p_{k}^{i}\;\right). \end{array}$$

To extract the most informative samples, the top *M* unlabelled samples with the maximum information entropy are selected and manually labelled. Then they are added to the initial training set. Since the certainty of the samples with small information entropy is high, the labels obtained by the CNN model are considered reliable. Therefore, *P* samples with small entropy are selected and labelled with the CNN. Then these samples are added to the initial training set as well. At this point, the training set is expanded.

Since the initial set of the labelled samples is small, the ability of a CNN model to measure the uncertainty of the samples is weak, which may cause a large deviation. Thus, instead of selecting *M* samples, it is more reasonable to select in an iterative manner. At the beginning of the training process, the epochs are small because the labelled samples are insufficient. With the increase of the training set, the epochs of the CNN can be increased gradually. In this paper, we select the epochs according to the following rule:

(8) $$\begin{array}{c} N=50+4i, \end{array}$$

where *i* denotes the iterations of the active learning algorithm and *N* denotes the epochs of the CNN during each iteration.

When a certain number of the unlabelled samples have been selected manually, the remaining samples are supposed to be reliable. If we continue selecting the unlabelled samples based on the active learning method, the improvement of generalization ability and the classification accuracy of the CNN is not ensured. Thus, after a certain number of the unlabelled samples are manually labelled, the iteration should be terminated. The main drawback of the CNN is that the training process costs too much time. In order to achieve a trade-off between the number of the unlabelled samples and the complexity of the algorithm, the iterations should be chosen appropriately.

### 3.2. Semisupervised Learning

Although the uncertainty of the remaining unlabelled samples is not significant, the class probability information contained in the samples can be used to improve the generalization ability of the CNN. We design a regularization term based on the class probability information of the unlabelled samples, which is added to the loss function of the CNN. The commonly used cost functions for the CNN include quadratic cost function, cross-entropy cost function, and log likelihood cost function. When the softmax layer is used as the output layer, the log likelihood cost function is simple and effective. The expression of the log likelihood cost function is as follows:

(9) $$\begin{array}{c} E_{0}=-\underset{x_{i}\in L}{\sum }\sum_{k}y_{k}^{i}\log\left(\;a_{k}^{i}\;\right)=-\underset{x_{i}\in L}{\sum }y^{i}\cdot \log\left(\;a^{i}\;\right), \end{array}$$

where *a*_*k*_^*i*^ represents the output of the *k*th neuron in the output layer corresponding to the input sample *x*_*i*_ and *y*_*k*_^*i*^ represents the expected value of the *k*th neuron. *y*^*i*^ and *a*^*i*^ denote the actual vector and the expected vector output from the softmax layer, respectively.

The maximum class probability output from the softmax layer is used to design the regularization term. The maximum output of the softmax layer relevant to the unlabelled input sample *x*_*i*_ is expressed as follows:

(10) $$\begin{array}{c} q_{max}^{i}=\max\left(\;\left[\;a_{1}^{i},a_{2}^{i},\ldots ,a_{N}^{i}\;\right]\;\right). \end{array}$$

The regularization term of the unlabelled samples is expressed by

(11) $$\begin{array}{c} E_{1}=-\underset{x_{i}\in u}{\sum }\sum_{k}q_{max}^{i}y_{k}^{i}\log\left(\;a_{k}^{i}\;\right), \end{array}$$

where *y*_*k*_^*i*^ denotes the label of the unlabelled samples assigned by the CNN. *q*_max_^*i*^ plays the role of a constraint. A higher value of *q*_max_^*i*^ indicates that the sample plays a more important role in the training process of the CNN model. After introducing the regularization term of the unlabelled samples, we have the cost function as follows:

(12) $$\begin{array}{c} E=E_{0}+b*E_{1}, \end{array}$$

where *b* is the weighting parameter.

For *E*_0_, the error vector of the output layer is

(13) $$\begin{array}{c} \delta_{0}^{L}=\frac{\partial E_{0}}{\partial z^{L}}=\underset{x_{i}\in L}{\sum }\left(\;a^{i}-y^{i}\;\right). \end{array}$$

For *E*_1_, the error vector of the output layer is

(14) $$\begin{array}{c} \delta_{1}^{L}=\frac{\partial E_{1}}{\partial z^{L}}=b\underset{x_{i}\in u}{\sum }q_{max}^{i}\left(\;a^{i}-y^{i}\;\right). \end{array}$$

According to (1), (13), and (14), the sensitivity of the modified cost function can be obtained as

(15) δ′δ0L+b∗δ1L=∑xi∈Lai−yi+b∑xi∈uqmaxiai−yi.

As the error vector of the output layer is obtained, the sensitivity of each layer can be calculated using (3) iteratively, and the parameters of each layer can be updated according to (4) and (5).

## 4. Experiment

**Figure 3:**
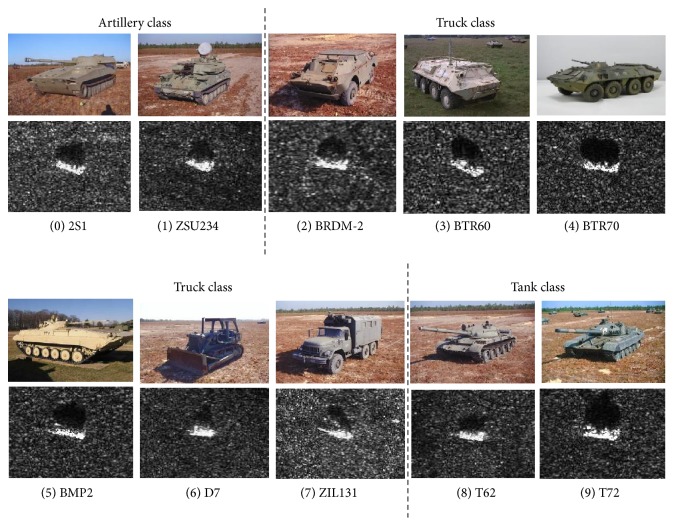
SAR images and corresponding optical images of ten types of targets in the MSTAR database.

We perform experiments on the Moving and Stationary Target Acquisition and Recognition (MSTAR) database, which is cofunded by National Defense Research Planning Bureau (DARPA) and the US Air Force Research Laboratory (AFRL). Ten types of vehicle targets in the MSTAR database are chosen in our experiment, that is, 2S1, ZSU234, BMP2, BRDM2, BTR60, BTR70, D7, ZIL131, T62, and T72. The SAR and corresponding optical images of each type are shown in Figure 3.  Table 1 lists the detailed information of target chips involved in this experiment.

### 4.1. The Effectiveness of the Active Learning Method

**Figure 4:**
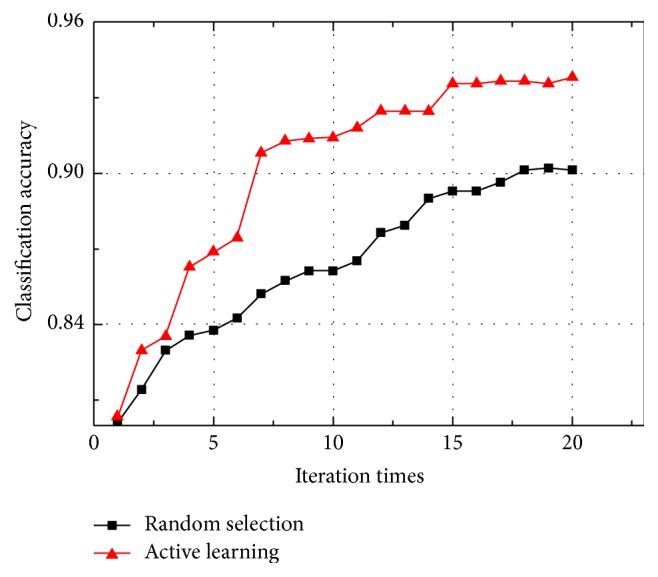
Classification accuracy of active learning method and random selection method.

We select 5% of the samples as the initial labelled training set which is used to train the CNN. Then the active learning method based on information entropy is utilized to expand the labelled training set in an iterative manner. During each iteration, the active learning method manually labels 8 samples with the largest information entropy, and 10 samples with the minimum information entropy are labelled by the CNN. To demonstrate the effectiveness of the active learning method, we compare it with the random selection method in which 8 unlabelled samples are randomly selected and manually labelled during each iteration. The classification accuracy of the above two methods varies with the iterations, as shown in Figure 4. Obviously, the classification accuracy of the active learning method is higher than that of the random selection method. Thus, it validates that the active learning method can select the unlabelled samples more effectively. The selected samples are then added to the labelled training set, which is helpful for improving the classification accuracy.

### 4.2. The Effectiveness of Semisupervised Learning Method

**Figure 5:**
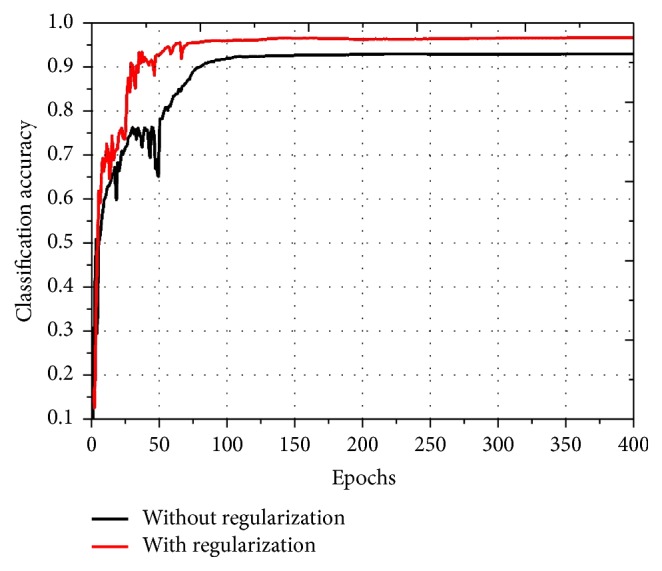
Classification accuracy of the methods with regularization and without regularization.

After the expansion of the initial labelled training set, the semisupervised learning method is utilized by obtaining the regularization term based on the remaining samples and we then add it to the cost function of the CNN. To demonstrate the effectiveness of the regularization method, we compare it with the method without any regularization. The comparison result is shown in Figure 5. As can be seen, the classification accuracy of the regularization method is higher than those without any regularization. The convergence speed of the regularization method is also faster because the method without any regularization needs 100 epochs for convergence, but the regularization method only needs 80 epochs. Thus, it validates that the regularization method is capable of utilizing the information contained in the unlabelled samples, which is helpful to improve the classification accuracy and the convergence speed of the proposed method.

### 4.3. Comparison with Other Methods

**Figure 6:**
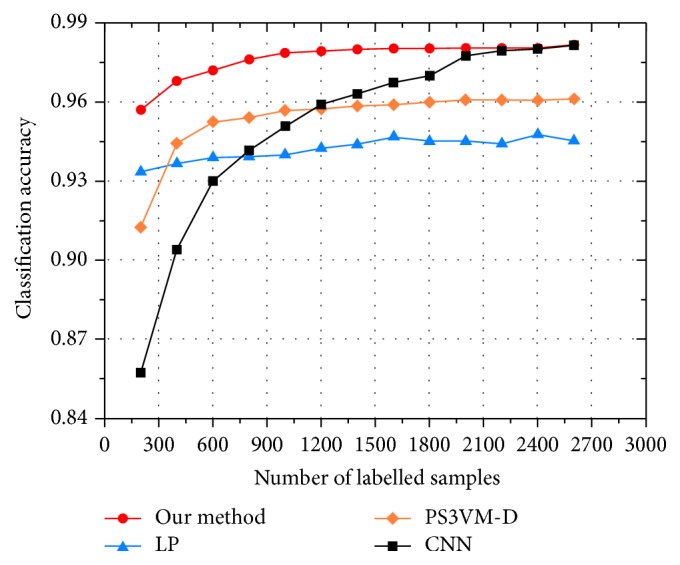
Classification accuracy of different classification methods.

In this section, we compare the performance of our method with that of the CNN [13], label propagation (LP) [19], and progressive semisupervised SVM with diversity (PS3VM-D) [18]. The CNN is a fully supervised algorithm which only utilizes the labelled samples to train the classification model. The LP and PS3VM-D are both semisupervised methods. The LP establishes a similar matrix and propagates the label of the labelled samples to the unlabelled samples according to the degree of similarity. The PS3VM-D selects the reliable unlabelled samples to expand the initial labelled training set. The comparison results are shown in Figure 6.

We find that our method outperforms the CNN method significantly when the labelled samples are insufficient, and this is due to our active learning and regularization strategy. The generalization ability of the CNN method is weak due to the small number of the labelled samples. With the increase of the labelled samples, the classification accuracy of the CNN method gradually increases, and ultimately it is the same as our method. Furthermore, the classification accuracy of our method is better than that of the other two semisupervised methods. The LP and PS3VM-D assign pseudolabels to the unlabelled samples; however, if the pseudolabels are wrong, the labels will have a negative influence on the subsequent classifier training process. In contrast, our method utilizes the unlabelled samples by designing a regularization term based on the class probability information; then the regularization term is added to the loss function of the CNN for the retraining purpose. In summary, our method is effective and has a strong generalization ability especially when the labelled samples are insufficient.

## 5. Conclusion

The CNN has achieved a great success in the field of image recognition. To improve the classification accuracy of the SAR image when the labelled samples are insufficient, a new active semisupervised CNN method has been proposed in this paper. First, we query the most informative and reliable samples by utilizing the active learning method, and then the semisupervised regularization is designed based on the remaining unlabelled samples. The main contributions of this paper are summarized as follows:

1. We used the active learning method to select the most informative and reliable samples, which are labelled manually and by the CNN, respectively. Thus, the overfitting issue is handled by adding the selected samples to the initial training set.
2. We designed a regularization term based on the class probability information of the unlabelled samples, and then the regularization term is added to the loss function of the CNN for retraining. Hence, the classification accuracy and generalization ability of the CNN were improved effectively.

From the experiment results, we observe that our method is effective and has a strong generalization ability especially when the labelled samples are insufficient; for example, the classification accuracy of our method is 95.7% when the number of the labelled samples is 236, which is apparently higher than the other methods using the same number of the labelled samples.

## Figures and Tables

**Table 1:** The training and testing set of our experiment.

Type	Tops	Model	Training set		Testing set	
			Depression	Number	Depression	Number
2S1	Artillery	B_01	17°	299	15°	274
ZSU234		D_08	17°	299	15°	274

BRDM2	Truck	E_71	17°	298	15°	274
BTR60		K10YT_7532	17°	256	15°	195
BMP2		SN_9563	17°	233	15°	195
BTR70		C_71	17°	233	15°	196
D7		92V_13015	17°	299	15°	274
ZIL131		E_12	17°	299	15°	274

T62	Tank	A_51	17°	299	15°	273
T72		#A64	17°	299	15°	274

			Sum: 2814		Sum: 2503	
